# Endoscopic Management of a Refractory Thigh Morel-Lavallée Lesion

**DOI:** 10.7759/cureus.93789

**Published:** 2025-10-03

**Authors:** Tiago P Barbosa, João C Mendes, Luís Fabião, Joana Azevedo, Tiago Frada

**Affiliations:** 1 Orthopaedics and Traumatology, Unidade Local Saude Braga, Braga, PRT; 2 Orthopaedics and Traumatology, Centro Hospital de Coimbra, Coimbra, PRT; 3 Orthopaedics and Traumatology, Unidade Local de Saúde de Barcelos/Esposende, Barcelos, PRT; 4 Orthopaedics and Traumatology, Hospital de Braga, Braga, PRT; 5 Orthopedics and Traumatology, Clínica Espregueira Mendes, Porto, PRT; 6 Orthopaedics and Traumatology, Hospital Lusíadas Braga, Braga, PRT

**Keywords:** endoscopic treatment, fibrin glue, mini-invasive technic, morel-lavallée lesion, percutaneous treatment

## Abstract

Morel-Lavallée lesions (MLLs) are post-traumatic closed degloving injuries characterized by the separation of skin and hypodermis from the underlying deep fascia, leading to the formation of fluid-filled cavities. We present the case of a 37-year-old male with a refractory distal thigh MLL sustained in a motor vehicle collision. After failed conservative management (aspiration and compression), the patient underwent endoscopic debridement with pseudocapsulectomy, drainage, and fibrin glue injection. Follow-up MRI at six months confirmed complete resolution with no recurrence. This approach combines the benefits of minimally invasive surgery, augmented with sclerotherapy, reducing the risk of complications, such as skin necrosis, when compared to open techniques because there is less recurrent aggression and promoting a faster recovery. While endoscopic techniques show promise for MLLs at high risk of wound complications, comparative studies are needed to establish optimal treatment algorithms.

## Introduction

Morel-Lavallée lesion (MLL) is a closed soft tissue degloving injury, typically caused by trauma, in which the skin and hypodermis are sheared from the underlying fascia, leading to the accumulation of fluid in the created dead space, due to the disruption of the perforating vessels and nerves and impaired lymphatic drainage [1,2]. Although traumatic injuries are common, these lesions are relatively rare and are frequently related to high-energy trauma, mainly motor vehicle collisions [1,3]. Due to the rarity of the injury, the exact incidence is difficult to determine; one retrospective series from Mayo Clinic, indicate 79 cases over eight years [4]. MLLs are frequently associated with specific injuries; their reported incidence in pelvic trauma ranges from 8.3% to 12.2% [4-6].

Often, MLL is not properly assessed or investigated upon admission due to coexisting injuries, typically more severe pelvic trauma and is frequently identified later through persistent seromas or during surgical procedures [3]. Nevertheless, MLLs pose significant therapeutic challenges. These lesions may become chronic, prove refractory to repeated interventions, and are frequently complicated by infections, often resulting in suboptimal functional and cosmetic outcomes [1].

Currently, there are no established management guidelines for MLLs. While several small cohort studies have examined treatment options, including conservative management, percutaneous aspiration, sclerodesis, and open surgery, their reported efficacy varies considerably [1,3].

This study aims to describe a minimally invasive endoscopic approach for the management of MLL and to evaluate its efficacy.

## Case presentation

**Figure 1:**
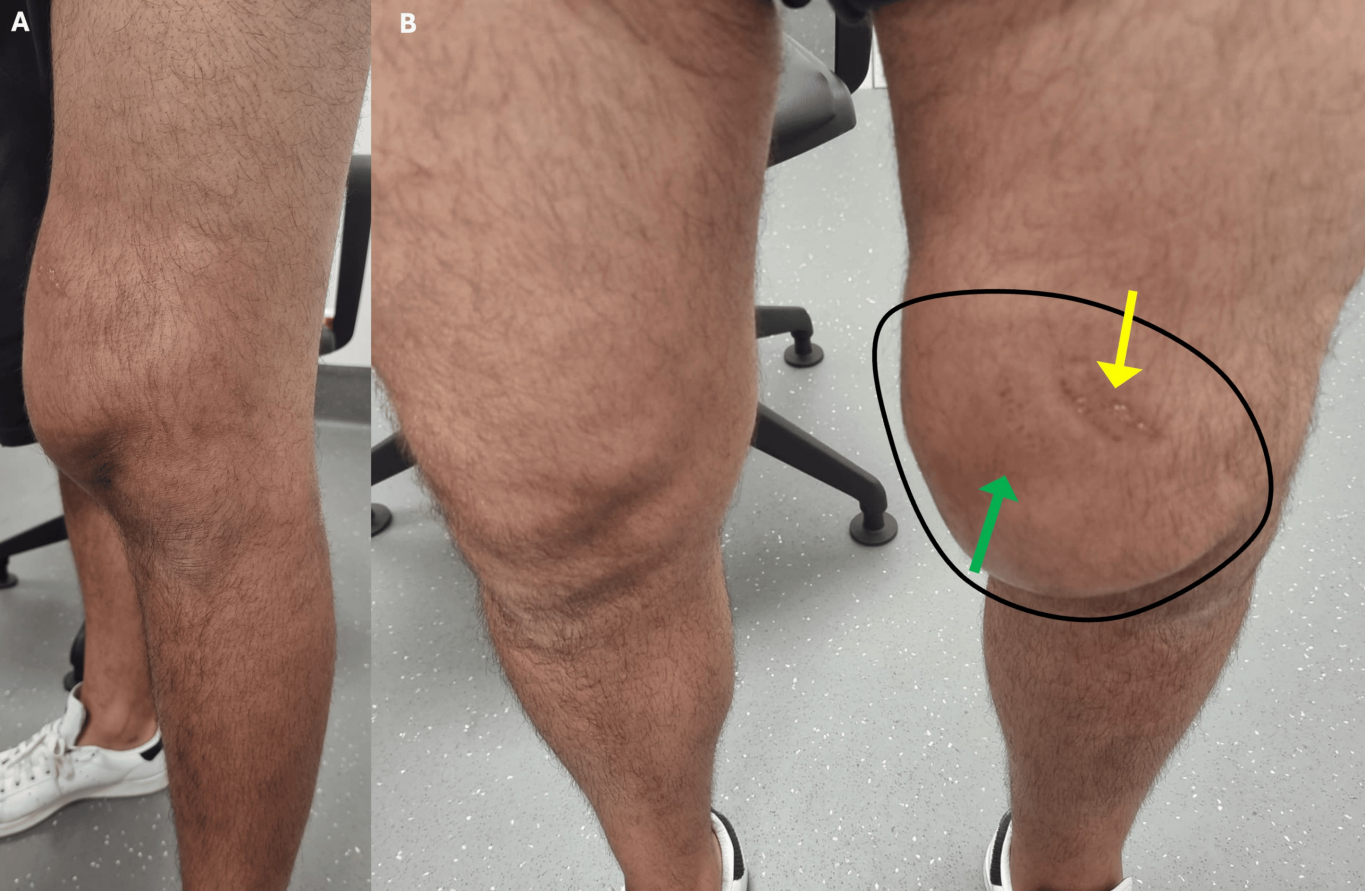
Swelling in the distal thigh (A) lateral view and (B) anterior view and comparison with contralateral. Black circle: Boundary of the swelling; Ecchymosis: Green arrow; Abrasions: Yellow arrow

**Figure 2:**
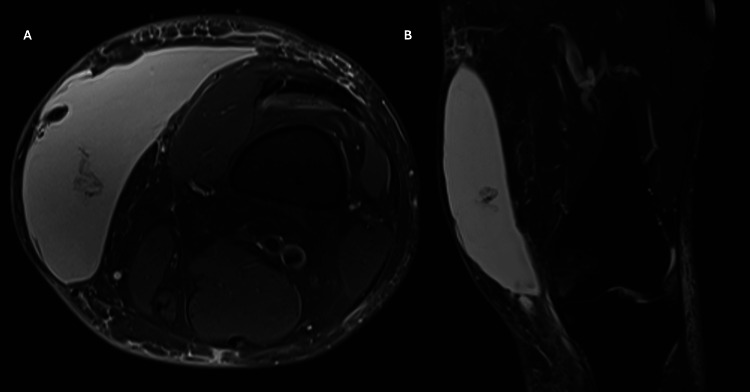
Non-contrast MRI axial view (A) and coronal view (B) of fluid collection

A 37-year-old male patient with no past medical history was evaluated in the emergency room (ER) of a general trauma hospital due to a pain in his left thigh after a traffic accident. The patient described that his knee was compressed between the steering wheel and the central console, and he managed to free himself with some effort. He was evaluated in the ER and presented only with local ecchymosis and abrasions of the thigh without gait impairment. After a normal X-ray, he was discharged with pain killers. However, a swelling in his distal thigh starts to develop gradually during one-two weeks after the traumatic event. Three weeks after the accident, the patient was referred to our hospital for evaluation. Physical examination revealed abnormal swelling and erythema on the medial distal thigh (compared with contralateral member) (Figure 1), with a soft palpable wave-like motion over the area. The patient was afebrile and without signs of cellulitis. Palpation revealed no fractures or crepitus, and the neurovascular exam was normal and symmetric. Fine needle aspiration yielded 500 mL of serosanguinous fluid, providing immediate symptomatic relief. Biochemical analysis of the fluid showed no leukocytic predominance or evidence of infection. However, the swelling recurred five days later. A non-contrast MRI was performed, revealing a fluid collection between the subcutaneous tissue and muscular fascia (Figure 2), leading to a diagnosis of an MLL.

**Figure 3:**
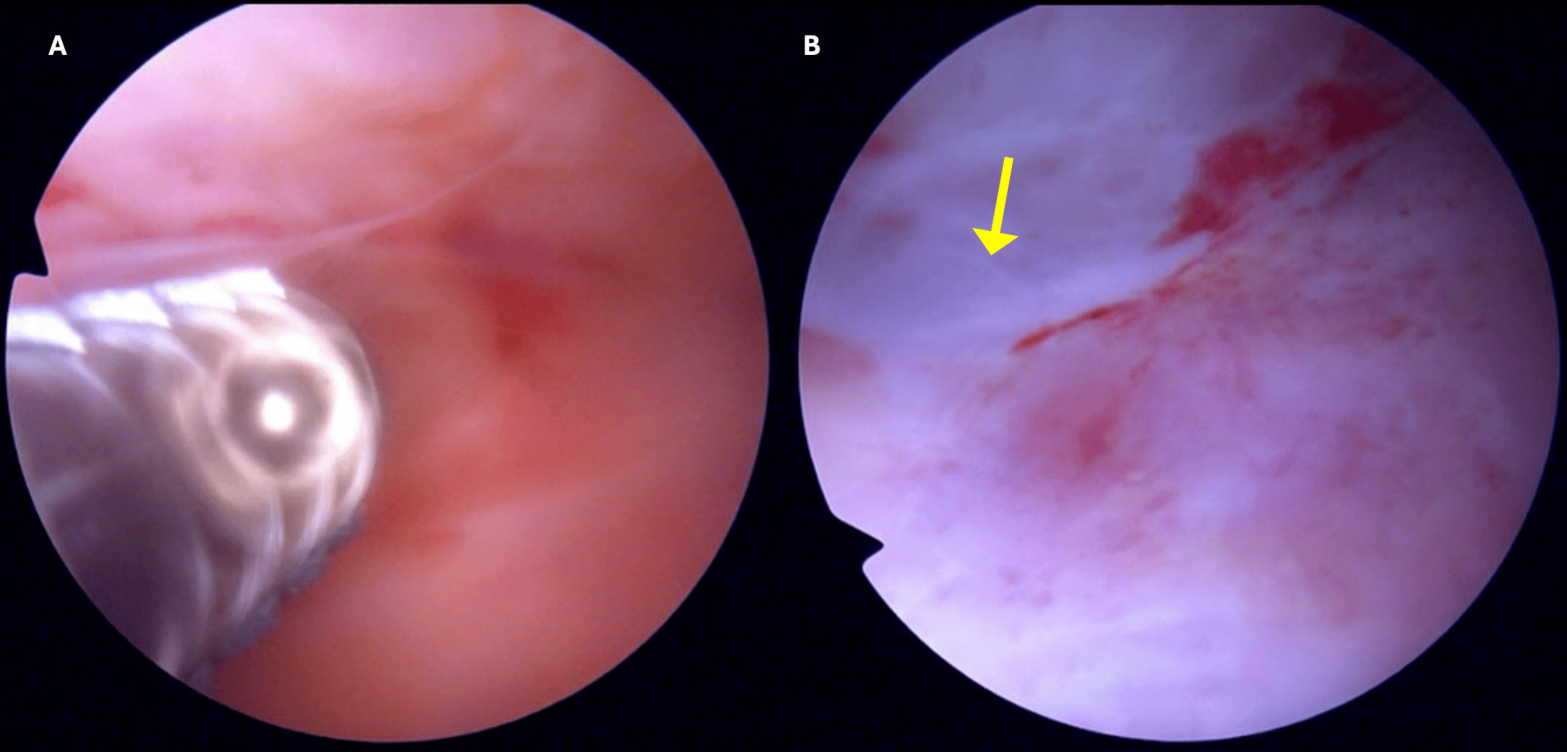
Endoscopic view of the reddish fluid (A) and white scar (yellow arrow) forming a pseudocapsule (B)

**Figure 4:**
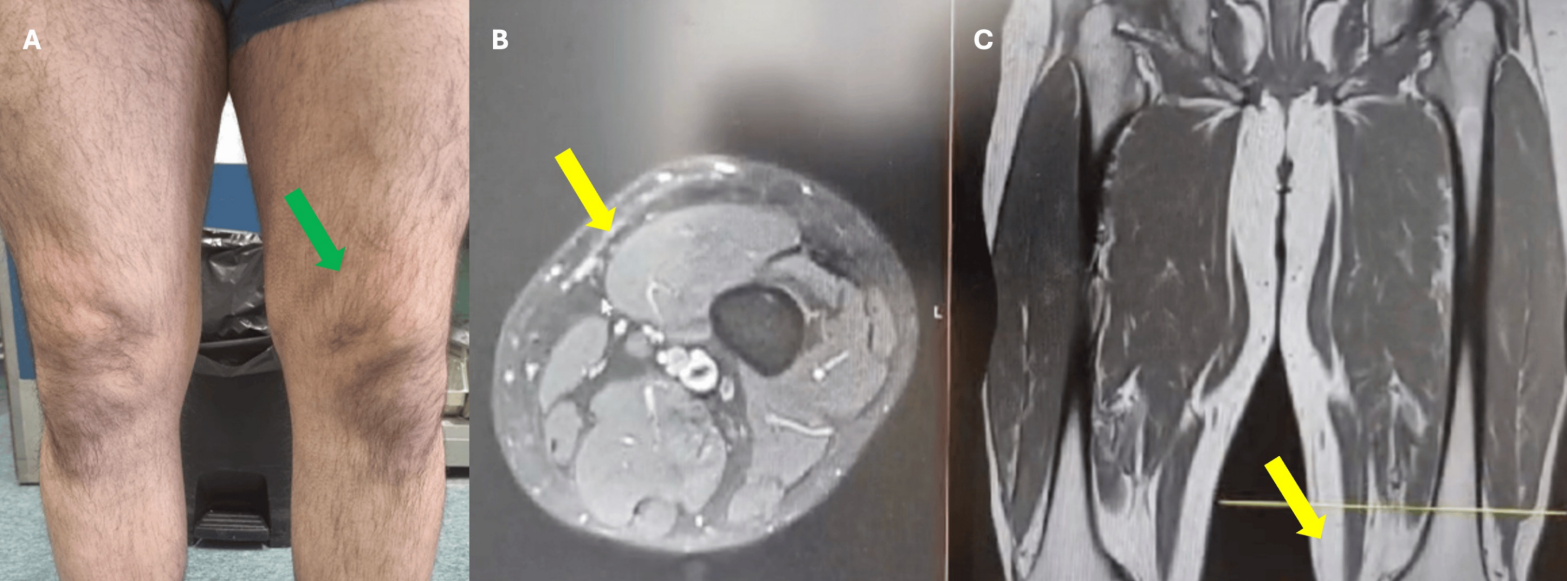
Six months after surgery without any swelling (green arrow) on the clinical observation (A) and with disappearance of the fluid collection on MRI (yellow arrows) axial view (B) and coronal view (C)

Following diagnosis, we attempted additional drainage procedures with compressive bandaging, but the lesion proved refractory to conservative treatment. Therefore, we decided to perform surgical treatment. Given the tissue's vulnerability from prior trauma, we pursued endoscopic management as the least invasive option to mitigate complication risks. The procedure was performed with the patient in the supine position under general anesthesia. Inspection was performed with a knee arthroscope with proximal and distal portals. The examination revealed an accumulation of reddish fluid and a white, scar-like pseudocapsule adherent to the tissue planes between the muscular fascia and the subcutaneous layer (Figure 3). This fibrous tissue was completely excised and debrided with a mechanical shaver and curettes to stimulate bleeding from the wound base. In addition, a dual vacuum drainage system was added postoperatively to optimize drainage. The drains were maintained for two weeks, and fibrin glue was injected at the time of drainage removal accompanied by a compression bandage. At outpatient follow-up, there was no evidence of recurrent fluid collection or surgical wound complications. Follow-up MRI at six months post surgery demonstrated complete healing with resolution of the thigh dead space and only residual scar tissue remaining (Figure 4). The patient made a full functional recovery, returning to work by the second postoperative month and subsequently resuming his regular sports activities without pain or functional limitations compared to his preinjury level.

## Discussion

MLLs result from shearing forces, most frequently affecting trunk and buttock regions, trochanteric region, anterolateral thigh, and knee [2,3]. While most cases occur post trauma, particularly motor vehicle accidents, sports-related injuries (e.g., football/soccer) represent another established etiology [1-3].

Diagnostic challenges arise from both the lesion’s nonspecific presentation (pain, inflammation, persistent swelling) and clinicians’ limited familiarity with MLLs, often leading to delayed or incorrect management [1,3]. The natural history of untreated MLLs may involve progression to localized infection or other chronic complications. [3].

While multiple treatment options for MLLs have been described in the literature, no standardized management protocol currently exists. Initial therapy typically involves conservative measures, including percutaneous aspiration with/without sclerotherapy and compressive dressings [1,3]. Beyond that, a systematic review data revealed significantly reduced healing times with surgical intervention versus compression therapy alone, and another study shows that lesions with a volume greater than 50 ml had a higher likelihood of recurring [4,7]. However, the presence of a well-formed pseudocapsule, which impedes fluid reabsorption, often renders conservative treatment ineffective [1,3,4,8,9]. In such refractory cases, surgical intervention becomes necessary.

Open drainage with debridement remains the standard surgical approach for MLLs. Critical to successful management is complete debridement of all devitalized tissue [1,3]. In MLLs featuring preserved overlying soft tissue and a well-formed pseudocapsule, total capsulectomy is normally necessary with additional tension suture techniques to minimize recurrence risk [1,3]. However, while effective, this technique carries inherent risks of functional impairment and surgical site infection [1,3].

Endoscopic techniques have been explored as a minimally invasive alternative to open surgical debridement for MLLs, aiming to reduce procedure-related morbidity [1,3]. Compared to conventional open approaches, endoscopic management offers distinct advantages including reduced tissue trauma, decreased postoperative pain, and accelerated recovery times [10-12].

The growing body of literature, including multiple published case series, has documented successful outcomes with endoscopic techniques. Pan et al. and Kon et al. reported resolution of MLL through percutaneous debridement augmented by fibrin glue instillation [13,14]. Similarly, Kim et al. reported successful resolution of MLL using endoscopic treatment combined with doxycycline sclerotherapy, and Liu et al. reported the same results with endoscopic debridement combined with percutaneous cutaneofascial suture technique [7,11]. Leng et al. presented the successful treatment results of a case series with 38 patients with a minimum follow-up of three years with just one complication [10]. Kage et al. reported their successful treatment of MLL with endoscopic debridement and negative pressure wound therapy [2]. Chan et al. reported resolution of a chronic MLL on the knee after endoscopic debridement [12]. In our case, endoscopic debridement and additional sclerotherapy were employed instead of other techniques. Current evidence supports sclerodesis as an effective treatment modality for MLLs up to 700 mL, with a pooled success rate of 95.7% across clinical series [3,9,15].

This study is limited by its nature as a single case report, which makes it difficult to establish standardized treatment protocols for the treatment of these lesions. A further limitation was our inability to evaluate the efficacy of isolated endoscopic treatment, as it was combined with an adjuvant application of fibrin glue.

This case report demonstrates successful endoscopic management of a distal thigh MLL. The minimally invasive approach was selected to reduce surgical morbidity and avoid wound-related complications. Adjuvant sclerodesis with fibrin glue was employed to enhance tissue healing and minimize recurrence risk.

## Conclusions

We recommend endoscopic debridement as an option for treatment approach for MLL, particularly in cases with an elevated risk of surgical wound complications. Additional procedures can be easily added to promote healing and prevent recurrence. However, further comparative studies are required to establish optimal treatment algorithms.
